# Conduction system pacing improves the outcomes on patients with high percentage of ventricular pacing and heart failure with mildly reduced ejection fraction

**DOI:** 10.3389/fcvm.2023.1132520

**Published:** 2023-05-16

**Authors:** Duo-duo Zhang, Fu-lu Zhao, Yi-heng Yang, Cheng-ming Ma, Pei-pei Ma, Yan-ni Zhao, Yun-long Xia, Lian-jun Gao, Ying-xue Dong

**Affiliations:** Department of Cardiology, The First Affiliated Hospital of Dalian Medical University, Dalian, China

**Keywords:** conduction system pacing, heart failure with mildly reduced ejection fraction, high percentage of ventricular pacing, his bundle pacing, left bundle branch pacing

## Abstract

**Aims:**

This study aimed to investigate the efficacy and safety of CSP in patients with a high percentage of ventricular pacing and heart failure with HFmrEF.

**Methods:**

Patients who underwent CSP for HFmrEF and ventricular pacing >40% were consecutively enrolled from January 2018 to May 2021. All participants were followed up at least 12 months. Clinical data including cardiac performance and lead outcomes were compared before and after the procedure. Left ventricular ejection fraction (LVEF) was measured using the biplane Simpson's method. HFmrEF was defined as heart failure with the LVEF ranging from 41%–49%.

**Results:**

CSP was successfully performed in 64 cases (96.97%), which included 16 cases of left bundle branch pacing (LBBP) and 48 cases of His bundle pacing (HBP). After a mean of 23.12 ± 8.17 months follow-up, NYHA classification (*P* < 0.001), LVEF (42.45 ± 1.84% vs. 49.97 ± 3.57%, *P* < 0.001) and left ventricular end diastolic diameter (LVEDD) (55.59 ± 6.17 mm vs. 51.66 ± 3.48 mm, *P* < 0.001) improved significantly. During follow-up, more than half (39/64,60.9%) of patients returned to normal LVEF and LVEDD with complete reverse remodeling. The pacing threshold in LBBP was lower (0.90 ± 0.27 V@0.4 ms vs. 1.61 ± 0.71 V@0.4 ms, *P *< 0.001) than that in HBP. No perforation, electrode dislodging, thrombosis or infection was observed during follow-up.

**Conclusions:**

CSP could improve the clinical outcomes in patients with HFmrEF and a high percentage of ventricular pacing. LBBP might be a better choice because of its feasibility and safety, especially in patients with infranodal atrioventricular block.

## 1. Introduction

Heart failure with mildly reduced ejection fraction (HFmrEF) occurs in 10%–20% of patients with heart failure (1, 2). Previous studies demonstrated that 17% of the patients with HFmrEF transitioned to HFrEF one year after follow-up, while 13% of the patients died during a median follow-up of 33 months (3). Furthermore, the prognosis of HFmrEF compared to HFrEF is worse than that of patients with stable HFrEF (4). HFmrEF is also associated with an increased risk of deterioration of cardiac function after right ventricular pacing (RVP). Previous trials, in which the patients with EF < 50% were included, had shown that cardiac resynchronization therapy (CRT) via biventricular pacing (BiVP) provided greater benefits than right ventricular pacing (5–7). Nevertheless, conventional BiVP may not be an ideal choice because of its limited response, more complicated procedure, and high cost (8).

Cardiac conduction system pacing (CSP), including His-bundle pacing (HBP) and left branch bundle pacing (LBBP), are promising alternatives to CRT (9, 10). In contrast to BiVP, CSP can maintain the electrical physiological conduction and left ventricular mechanical synchronization. However, it is not clear until now whether CSP is the optimal option for patients with HFmrEF and a high percentage of ventricular pacing. This study aimed to explore the clinical performance and safety of CSP in patients with HFmrEF and a high percentage of ventricular pacing.

## 2. Methods

### 2.1. Patients' enrollment and follow-up

Patients with HFmrEF and CSP for the ventricular pacing >40% were consecutively enrolled from January 2018 to June 2021. Regular follow-up was conducted at 1, 3, 6, 12,18 and 24 months postoperatively. All the patients met the criteria of complete, high-grade and second-degree of Mobitz type II AVB, and the ventricular pacing >40% was verified by pre-operation prediction and post-operation follow-up. Exclusion criteria were recent myocardial infarction, device upgrade, QRS complex >130 ms, AV node ablation, and recent cardiac surgery (<3 months). LBBP would be the alternative therapy in patients with infranodal atrioventricular (AV) block, or the first choice of failed HBP, and BiVP would be the rescue therapy if CSP failed.

During follow-up, symptoms of heart failure, echocardiography, 12-lead electrocardiogram (ECG), pacemaker parameters, and postoperative complications were monitored in the outpatient clinic. Maximum mitral regurgitation (MR) and tricuspid regurgitation (TR) were measured using the vena contracta width with color-flow Doppler, and Left ventricular ejection fraction (LVEF) was measured using the biplane Simpson's method.

### 2.2. Criteria and definition

HFmrEF was defined as heart failure with the LVEF ranging from 41%–49%. Ventricular pacing >40% was considered as a high percentage of ventricular pacing. CRT response was defined as a relative increase (≥15%) or absolute increase (≥10%) in LVEF after one year. Stim-left ventricular active time (LVAT) less than 75 ms, an abrupt decrease in LVAT of longer than 10 ms and the morphologies of Qr, qR, or rSR’ in lead V_1_ were the simple criteria for left bundle branch capture. Discrete local ventricular potential appears during selective LBBP indicating that local myocardium was no longer captured, however, not all patients demonstrated the selective LBBP. HBP was accepted when capture threshold was lower than 2.0 V/0.4 ms and the amplitude of *R* wave was higher than 4.0 mV in patients with acceptable His–ventricular conduction.

### 2.3. CSP procedure and device programming

HBP and LBBP were performed using the Select Secure pacing lead (Model 3830, 69 cm, Medtronic Inc., Minneapolis, MN, USA) and a fixed-curve sheath (C315 HIS, Medtronic Inc.). His bundle electrograms were mapped in a unipolar configuration and recorded in a system (Prucka Cardiolab, GE Healthcare, Waukesha, WI), as described in our previous publications (11). HBP was not considered if 1:1 His–ventricular conduction was not demonstrable during pacing at a rate of 120 beats per minute in patients with infranodal AV block. LBBP was further performed in these patients or HBP failed. The unipolar tip-paced QRS configuration and pacing impedance were monitored along with the measurement of left ventricular activation time in lead V_5_. For patients with HBP or with ventricular pacing dependence and an escape rate less than 40 beats per minute, right ventricular backup pacing was performed.

The 3830 lead was connected to the left ventricular (LV) port in patients with right ventricular (RV) lead backup, and the LV-RV delay was programmed to ensure the shortest QRS duration. No atrial leads were placed for patients with a left atrial diameter (LAD) >50 mm, atrial fibrillation (AF) lasting >5 years, and no expectation of sinus rhythm. In patients with permanent AF who required ventricular pacing backup, the 3,830 lead was connected to the right atrial (RA) port and the right ventricular lead remained in the RV port, and a shorter AV interval and blanking period were programmed to ensure minimal ventricular pacing.

If CSP was unsuccessful, an LV lead was implanted using a consecutive coronary venous approach. In patients with BiVP, the LV lead was positioned using a standard technique in the lateral or posterolateral LV vein. An RV lead was implanted into the right ventricular septum.

### 2.4. Statistical analysis

Categorical variables were expressed as numbers (%) and compared using Fisher's exact test. Continuous variables were expressed as the mean ± SD or median and were compared with independent two-sample, Wilcoxon test, or paired *t* test. *P *< 0.05 (two-tailed) was considered statistically significant. SPSS25.0 software was used for statistical analysis.

## 3. Results

### 3.1. Baseline patient characteristics and clinical events

A total of 66 patients underwent device implantation, including 48 patients with HBP, with a success rate of 81.28%; 16 patients (10 patients with infranodal AV block and failed in 1:1 His–ventricular conduction at a pacing rate of 120 bpm) with LBBP with a success rate of 93.75%; and 2 patients with BiVP for failed CSP. Among the 16 patients with LBBP, 10 patients demonstrated with selective LBBP. RV backup pacing lead was implanted in all patients with HBP and eight patients (50.00%) with LBBP. All patients were followed up for 23.12 ± 8.17 months. No infection, thrombosis, acute left heart failure, perforation, lead dislodging, or sudden death was observed. Four patients with CSP were re-hospitalized, and one patient died of kidney failure approximately two years after the operation.

There were no significant differences in sex, age, B-type natriuretic peptide level, ECG characteristics, and comorbidity between patients with LBBP and HBP (*P *> 0.05) (Supplementary Table S1).

### 3.2. Clinical outcomes after CSP

Approximately 85.94% (55/64) of the patients responded to CSP. Complete LV reverse remodeling was observed in 39 patients (60.94%). Improvements in cardiac remodeling and cardiac performance are shown in Supplementary Table S2. LVEF (42.45 ± 1.84% vs. 49.97 ± 3.57%, *P* < 0.001), NYHA classification (*P* < 0.001), LVEDD (55.59 ± 6.17 mm vs. 51.66 ± 3.48 mm, *P* < 0.001) and LAD (47.13 ± 5.87 mm vs. 43.84 ± 5.43 mm, *P* < 0.001) improved significantly. QRS duration (106.83 ± 10.23 ms vs. 108.50 ± 9.69 ms, *P *= 0.201) showed no significant changes after CSP procedure. No patients deteriorated to moderate/severe MR or TR.

### 3.3. Lead outcome after CSP

The pacing percentage at the final follow-up was 82.27 ± 23.80%. The threshold of CSP remained stable (1.32 ± 0.59 V@0.4 ms vs. 1.50 ± 0.71 V@0.4 ms, *P *= 0.27) after follow-up. Impedance showed a significant decrease after follow-up (726.94 ± 200.50 *Ω* vs. 492.94 ± 146.51 *Ω*, *P *< 0.001). All the changes are shown in Supplementary Table S3. The threshold increased obviously (3.0 V@1.0 ms) in one patient one year after HBP, and then decreased to 1.0 V@0.4 ms and remained stable after resetting the lead to LBBP modality.

### 3.4. Different outcomes between HBP and LBBP

The QRS duration was a little shorter (107.08 ± 10.04 ms vs. 112.75 ± 7.26 ms, *P *= 0.04) and pacing threshold was a little higher (1.61 ± 0.71 V@0.4 ms vs. 0.90 ± 0.27 V@0.4 ms, *P *< 0.001) in patients with HBP as compared to patients with LBBP. However, LVEF (49.85 ± 3.96% vs. 50.31 ± 2.09%, *P *= 0.66), LAD (43.44 ± 4.69 mm vs. 45.06 ± 7.26 mm, *P *= 0.30) and LVEDD (52.02 ± 3.76 mm vs. 50.31 ± 2.09 mm, *P *= 0.15) were not significantly different between HBP and LBBP after follow-up.

The pacing threshold (1.61 ± 0.71 V@0.4 ms vs. 0.90 ± 0.27 V@0.4 ms, *P *< 0.001) and amplitude of *R* wave (4.28 ± 3.67 vs. 14.82 ± 7.19, *P *= 0.004) were different in patients with HBP and in those with LBBP.

## 4. Discussion

We demonstrated that CSP could improve the clinical performance in patients with HFmrEF and ventricular pacing >40%. We also showed that LBBP was a favorable CSP modality, especially for those with infranodal AV block.

### 4.1. Feasibility and safety of CSP on patients with hFmrEF and AVB

This study revealed that the success rate of permanent CSP was as high as 97.0% in the patients with HFmrEF and ventricular pacing >40%. While the reported failure rate of BiVP was only 3.6%, Dr. Gamble et al. noted that the suboptimal position was accepted in 20% patients, which would be less likely to benefit patients who underwent BiVP therapy (12). Bhatt et al. reported that 8% patients with HBP required electrode adjustment (13). However, our study demonstrated stable and acceptable thresholds for HBP one year after operation (14). In our study, only one patient with HBP underwent electrode adjustment one year after the operation. The lower electrode adjustment might be due to the strict criterion for HBP with a capture threshold lower than 2.0 V@0.4 ms and an amplitude higher at 4 mV in our study. By virtue of being in the septal myocardium, the distal HBP lead helix plays an important role in the favorable capture threshold and amplitude (15).

However, the failure of HBP is not a negligible issue (16). Vijayaraman et al. reported 84% success rate for HBP in unselected patients with AV block (93% AV nodal and 76% infranodal) (17). Possible reasons for this include the relatively high threshold of HBP, block position, and block progression. To utilize HBP first and then go to LBBP might no longer be the case in contemporary practice. LBBP was associated with a higher success rate and lower pacing thresholds than HBP (18, 19). In our study, LBBP also showed a promising modality for better pacing variables of threshold (0.90 ± 0.27 V@0.4 ms) and amplitude (14.82 ± 7.19 mV) than HBP. Ten patients were detected with clinical infranodal AV block for 1:1 His–ventricular conduction at a pacing rate of 120 bpm, which was not available in our study, suggesting that LBBP was indispensable for a broader range of applications on the infranodal conduction block.

### 4.2. Clinical performance of CSP on patients with hFmrEF and AVB

Since the Block-HF test (20), HOBIPACE test (21) and COMBAT test (22), the benefits of BiVP on LV reverse remodeling and clinical outcomes in mild to severe HF patients with AV block and LVEF <50% have been well known. However, it is difficult to reverse remodeling completely for impaired conduction defects (9). Furthermore, patients with HFmrEF were enrolled in previous studies. Kanai et al. reported that a significant improvement in LVEF was not observed after *de novo* BiVP in patients with HFmrEF and AV block (23). The PACE trial, which enrolled HF patients with LVEF <45% and AV block, demonstrated no significant change in LVESV and LVEF over two years in patients with BiVP (24). Albertsen et al. reported a small randomized controlled trial, which included HFmrEF patients with AVB, and showed that LVEF and LVESV were preserved rather than improved one year after BiVP (25).

We showed that the response rate was 85.94%, and 60.94% patients with normalized LVEF and LVEDD after CSP. LVEDD decreased from 55.59 ± 6.17 mm to 51.66 ± 3.48 mm (*P *< 0.001). LVEF improved from 42.45 ± 1.84% to 49.97 ± 3.57% (*P *< 0.001), and the NYHA also improved significantly (*P* < 0.001). Compared to the previous studies on patients with BiVP for AV block and LVEF <50% (20–22), the key point resulting in this favorable outcome might be that the QRS duration did not get prolonged significantly (106.83 ± 10.23 ms vs. 108.50 ± 9.69 ms, *P *= 0.20) after CSP. QRS duration and morphology reflect the electrical timing and activation sequence of the ventricles. The more synchrony there is, the higher the likelihood of a favorable outcome (26–28). Better electrical synchrony due to CSP might be the reason why CSP could improve or preserve cardiac performance in patients with HFmrEF and AV block. A meta study with 546 patients demonstrated that HBP improved TR grade after HBP for CRT and AVB (29). Dr. Tung et al. also found that HBP reduced functional MR through favorable ventricular remodeling in patients with LV systolic dysfunction (30). Dr. Vijayaraman et al. showed that LBBP resulted in excellent electrical resynchronization and no worsening of functional MR from baseline in all 73 patients (31). Consistent with those studies, no patients deteriorated to moderate/severe MR or TR in this current study. However, more studies would be required to investigate the effect of CSP on tricuspid valve and mitral valve in the future.

### 4.3. Different options for cardiac resynchronization in patients with HFmrEF and AV block

BLOCK-HF was the first larger trial to show the clinical benefit of the BiVP in patients with reduced LVEF (<50%) and AV block (20). However, the BLOCK-HF study included patients with a wide QRS and reduced LV function, both of whom were excluded from our study.

Recently, several conduction system pacing modalities such as His optimized CRT, LBBP, and HBP pacing have been developed as alternative pacing methods for RV pacing in patients with AV block, and the studies demonstrated that LVEF in RVP group was significantly lower than HBP especially in patients with AV block and LVEF >40% (32–34). Our results also demonstrate that CSP might be a priority recommendation for all patients with LVEF <50% and ventricular pacing dependence.

It is very important that we found that the improvement in LVEF after LBBP was not different after HBP during the follow-up (50.31 ± 2.09% vs. 49.85 ± 3.96%, *P *= 0.66) in patients with HFmrEF diagnosed for the first time. Furthermore, we also demonstrated that the threshold was lower (0.90 ± 0.27 V@0.4 ms vs. 1.61 ± 0.71 V@0.4 ms, *P *< 0.001) in LBBP than in HBP after follow-up. Reticular distribution of the left conduction bundle may provide a better anatomical basis for LBBP. Compared with the His bundle, it provides a wider pacing space and a higher success rate (35). Additionally, LBBP can capture the myocardium around the conduction bundle at low voltage when pacing the left bundle branch, and it might be helpful in preventing the possible dangers caused by the progression of conduction system lesions.

An obvious improvement in cardiac performance was observed in patients with HFmrEF after CSP. The physiological electrical conduction resulting from the CSP procedure played a major role in the favorable outcome; however, the benefit from drug treatment, including β-blockers, after CSP might partly contribute to the favorable results. Additionally, since LV dysfunction might be partly secondary to AV block and bradycardia, it was more likely to be reversed with CSP.

### 4.4. Limitations

Due to the lack of randomized controlled trials, it is impossible to draw a direct conclusion that CSP is superior to BiVP in patients with HFmrEF and AV block. It is necessary to conduct randomized controlled clinical trials with more samples to further confirm these findings and provide evidence for the widespread promotion of HPCSP in the future.

## 5. Conclusion

HPCSP improved the clinical outcomes in patients with HFmrEF and a high percentage of ventricular pacing. LBBP may be a better choice because of its feasibility and safety, especially in patients with clinical infranodal atrioventricular block.

## Data Availability

The original contributions presented in the study are included in the article/**Supplementary Material**, further inquiries can be directed to the corresponding author.
