# Supplementary material for: Conduction system pacing improves the outcomes on patients with high percentage of ventricular pacing and heart failure with mildly reduced ejection fraction
Source: Front Cardiovasc Med. 2023 May 16;10:1132520. doi: 10.3389/fcvm.2023.1132520 (PMC10228713; doi:10.3389/fcvm.2023.1132520)
Supplement: Supplementary file 1 [file Table1.docx]

Supplementary Material

# Supplementary Table

Table 1. Basic characteristics of patients with CSP

|  | **Total patients**  **(n=64)** | **HBP patients (n=48)** | **LBBP patients (n=16)** | ***P*** |
| --- | --- | --- | --- | --- |
| Male (n, %) | 43(67.19%) | 34(70.83%) | 9(56.25%) | 0.28 |
| Age (years) | 75.14±9.00 | 75.38±9.80 | 74.44±6.26 | 0.66 |
| NYHA classification | 3(3,3) | 3(3,3) | 3(2,3) | 0.82 |
| LVEF (%) | 42.45±1.84 | 42.40±1.90 | 42.63±1.71 | 0.67 |
| BMI (kg/m2) | 26.44±4.53 | 26.52±4.72 | 26.20±4.08 | 0.81 |
| BNP (ng/L) | 452.45±226.21 | 456.72±235.84 | 438.30±198.95 | 0.80 |
| Crea (μmol/L) | 91.17±26.99 | 92.74±29.38 | 86.69±18.57 | 0.44 |
| QRS duration (ms) | 106.83±10.23 | 106.60±11.30 | 107.50±6.22 | 0.70 |
| MR grade |  |  |  |  |
| Mild (n, %) | 34(53.13%) | 26(54.17%) | 8(50.00%) | 0.11 |
| Moderate (n, %) | 18(28.13%) | 13(27.08%) | 5(31.25%) | 0.75 |
| Severe (n, %) | 3(4.69%) | 2(4.17%) | 1(6.25%) | 0.73 |
| TR grade |  |  |  |  |
| Mild (n, %) | 29(45.31%) | 24(50.00%) | 9(56.25%) | 0.67 |
| Moderate (n, %) | 17(26.56%) | 10(20.83%) | 7(43.75%) | 0.07 |
| Severe (n, %) | 3(4.69%) | 2(4.17%) | 1(6.25%) | 0.73 |
| LVEDD (mm) | 55.59±6.17 | 55.98±6.05 | 54.44±6.56 | 0.39 |
| LAD (mm) | 47.13±5.87 | 47.08±6.09 | 47.25±5.35 | 0.92 |
| Diabetes mellitus (n, %) | 13(20.31%) | 8(16.67%) | 5(31.25%) | 0.21 |
| Hypertension (n, %) | 32(50.00%) | 24(50.00%) | 8(50.00%) | 1.00 |
| Chronic kidney disease (n, %) | 7(10.94%) | 6(12.50%) | 1(6.25%) | 0.49 |
| Coronary heart disease (n, %) | 21(32.81%) | 15(31.25%) | 6(37.50%) | 0.65 |
| Ventricular tachycardia/fibrillation (n, %) | 5(7.81%) | 3(6.25%) | 2(12.50%) | 0.42 |
| Atrial fibrillation (n, %) | 10(15.63%) | 7 (17.07%) | 3(18.75%) | 0.69 |
| ARNI/ACEI/ARB | 29 (45.31%) | 22 (45.83%) | 7(43.75%) | 0.89 |
| Diuretics (n, %) | 34(53.13%) | 27 (56.25%) | 7(43.75%) | 0.39 |
| Spironolactone (n, %) | 34(53.13%) | 27 (56.25%) | 7(43.75%) | 0.39 |
| Statins (n, %) | 28(43.75%) | 18 (37.50%) | 10(62.50%) | 0.08 |
| Aspirin (n, %) | 18(28.13%) | 15 (31.25%) | 3(18.75%) | 0.34 |
| Nitrates (n, %) | 19(29.69%) | 16(33.33%) | 3(18.75%) | 0.27 |

*ACEI* angiotensin converting enzyme inhibitors; *ARB* angiotensin receptor blocker; *ARNI* angiotensin receptor neprilysin inhibitors; *BNP* B-type Natriuretic Peptide; *BMI* body mass index; *HBP* His bundle pacing; *CSP* conduction system pacing; *LAD* left atrial diameter; *LVEDD* left ventricular end-diastolic diameter; *LVEF* left ventricular ejection fraction; *MR* mitral regurgitation; *TR* tricuspid regurgitation.

Table 2. Clinical outcomes of HBP and LBBP

|  | **Total patients (n=64)** | **HBP patients (n=48)** | **LBBP patients (n=16)** | ***P*** |
| --- | --- | --- | --- | --- |
| initial QRS d (ms) | 106.83±10.23 | 106.60±11.30 | 107.50±6.22 | 0.70 |
| final QRS d (ms) | 108.50±9.69 | 107.08±10.04 | 112.75±7.26 | 0.04 |
| initial NYHA classification | 3(3,3) | 3(3,3) | 3(2,3) | 0.85 |
| final NYHA classification | 1(1,2)* | 1(1,2)* | 1(1,1.8)* | 0.17 |
| initial LAD (mm) | 47.13±5.87 | 47.08±6.09 | 47.25±5.35 | 0.92 |
| final LAD (mm) | 43.84±5.43* | 43.44±4.69* | 45.06±7.26* | 0.30 |
| initial LVEDD (mm) | 55.59±6.17 | 55.98±6.05 | 54.44±6.56 | 0.39 |
| final LVEDD (mm) | 51.66±3.48* | 52.02±3.76* | 50.31±2.09* | 0.15 |
| initial LVEF (%) | 42.45±1.84 | 42.40±1.90 | 42.63±1.71 | 0.67 |
| final LVEF (%) | 49.97±3.57* | 49.85±3.96* | 50.31±2.09* | 0.66 |
| initial MR grade, mild (n, %) | 34(53.13%) | 26(54.17%) | 8(50.00%) | 0.11 |
| final MR grade, mild (n, %) | 20 (31.25%)* | 18 (45.83%)* | 2 (31.25%)* | 0.18 |
| initial TR grade, mild (n, %) | 29(45.31%) | 24(50.00%) | 9(56.25%) | 0.67 |
| final TR grade, mild (n, %) | 17 (26.56%)* | 14 (29.17%)* | 3 (18.75%)* | 0.41 |

*LAD* left atrial diameter; *LVEDD* left ventricular end-diastolic diameter; *LVEF* left ventricular ejection fraction; *MR* mitral regurgitation; *QRS d*, QRS duration; *TR* tricuspid regurgitation; **P*<0.05, compared to before surgery

Table 3. Lead outcomes of HBP and LBBP

|  | **Total patients (n=64)** | **HBP patients (n=48)** | **LBBP patients (n=16)** | ***P*** |
| --- | --- | --- | --- | --- |
| VP percentage (%) | 82.27±23.80 | 82.98±24.56 | 80.23±22.08 | 0.69 |
| initial threshold (V@0.4ms) | 1.32±0.59 | 1.42±0.63 | 1.00±0.31 | 0.001 |
| final threshold (V@0.4ms) | 1.50±0.71 | 1.61±0.71 | 0.90±0.27 | <0.001 |
| initial amplitude of R wave (mV) | 6.24±4.73 | 5.15±3.49 | 9.76±6.43 | 0.02 |
| final amplitude of R wave (mV) | 5.91±5.76 | 4.28±3.67 | 14.82±7.19 | 0.004 |
| initial impedance(Ω) | 726.94±200.50 | 712.38±211.68 | 770.63±160.41 | 0.32 |
| final impedance(Ω) | 492.94±146.51* | 484.93±144.61* | 539.00±158.84* | 0.34 |

*VP*: ventricular pacing, **P<*0.05, compared with before operation
